# Cross-ethnic Molecular Signatures Underpin the Adverse Impact of Statin Use on Type 2 Diabetes

**DOI:** 10.1093/gpbjnl/qzaf101

**Published:** 2025-11-06

**Authors:** Fengzhe Xu, Min Yang, Wei Hu, Shuai Yuan, Xue Cai, Wanglong Gou, Zelei Miao, Bang-yan Li, Liang Yue, Zhangzhi Xue, Menglei Shuai, Luqi Shen, Yuanqing Fu, Tiannan Guo, Yu-ming Chen, Ju-Sheng Zheng

**Affiliations:** Westlake Center for Intelligent Proteomics, Westlake Laboratory of Life Sciences and Biomedicine, Hangzhou 310024, China; Westlake Center for Intelligent Proteomics, Westlake Laboratory of Life Sciences and Biomedicine, Hangzhou 310024, China; Guangdong Provincial Key Laboratory of Food, Nutrition and Health, Department of Epidemiology, School of Public Health, Sun Yat-sen University, Guangzhou 510275, China; Unit of Cardiovascular and Nutritional Epidemiology, Institute of Environmental Medicine, Karolinska Institutet, Stockholm 17177, Sweden; Westlake Center for Intelligent Proteomics, Westlake Laboratory of Life Sciences and Biomedicine, Hangzhou 310024, China; School of Medicine and School of Life Sciences, Westlake University, Hangzhou 310024, China; Institute of Basic Medical Sciences, Westlake Institute for Advanced Study, Hangzhou 310024, China; Westlake Center for Intelligent Proteomics, Westlake Laboratory of Life Sciences and Biomedicine, Hangzhou 310024, China; School of Medicine and School of Life Sciences, Westlake University, Hangzhou 310024, China; Institute of Basic Medical Sciences, Westlake Institute for Advanced Study, Hangzhou 310024, China; Westlake Center for Intelligent Proteomics, Westlake Laboratory of Life Sciences and Biomedicine, Hangzhou 310024, China; School of Medicine and School of Life Sciences, Westlake University, Hangzhou 310024, China; Institute of Basic Medical Sciences, Westlake Institute for Advanced Study, Hangzhou 310024, China; Guangdong Provincial Key Laboratory of Food, Nutrition and Health, Department of Epidemiology, School of Public Health, Sun Yat-sen University, Guangzhou 510275, China; Westlake Center for Intelligent Proteomics, Westlake Laboratory of Life Sciences and Biomedicine, Hangzhou 310024, China; School of Medicine and School of Life Sciences, Westlake University, Hangzhou 310024, China; Institute of Basic Medical Sciences, Westlake Institute for Advanced Study, Hangzhou 310024, China; Westlake Center for Intelligent Proteomics, Westlake Laboratory of Life Sciences and Biomedicine, Hangzhou 310024, China; School of Medicine and School of Life Sciences, Westlake University, Hangzhou 310024, China; Institute of Basic Medical Sciences, Westlake Institute for Advanced Study, Hangzhou 310024, China; Westlake Center for Intelligent Proteomics, Westlake Laboratory of Life Sciences and Biomedicine, Hangzhou 310024, China; School of Medicine and School of Life Sciences, Westlake University, Hangzhou 310024, China; Westlake Center for Intelligent Proteomics, Westlake Laboratory of Life Sciences and Biomedicine, Hangzhou 310024, China; School of Medicine and School of Life Sciences, Westlake University, Hangzhou 310024, China; Institute of Basic Medical Sciences, Westlake Institute for Advanced Study, Hangzhou 310024, China; Westlake Center for Intelligent Proteomics, Westlake Laboratory of Life Sciences and Biomedicine, Hangzhou 310024, China; School of Medicine and School of Life Sciences, Westlake University, Hangzhou 310024, China; Institute of Basic Medical Sciences, Westlake Institute for Advanced Study, Hangzhou 310024, China; Westlake Center for Intelligent Proteomics, Westlake Laboratory of Life Sciences and Biomedicine, Hangzhou 310024, China; School of Medicine and School of Life Sciences, Westlake University, Hangzhou 310024, China; Institute of Basic Medical Sciences, Westlake Institute for Advanced Study, Hangzhou 310024, China; Guangdong Provincial Key Laboratory of Food, Nutrition and Health, Department of Epidemiology, School of Public Health, Sun Yat-sen University, Guangzhou 510275, China; Westlake Center for Intelligent Proteomics, Westlake Laboratory of Life Sciences and Biomedicine, Hangzhou 310024, China; School of Medicine and School of Life Sciences, Westlake University, Hangzhou 310024, China; Institute of Basic Medical Sciences, Westlake Institute for Advanced Study, Hangzhou 310024, China

**Keywords:** Statin, T2D, Multi-omics, Cross-ethnic, Mendelian randomization

## Abstract

The use of statins as the primary therapy for reducing low-density lipoprotein cholesterol has raised concerns regarding their potential side effects in increasing the risk of type 2 diabetes (T2D). However, the underlying mechanism remains largely unknown. In this study, we utilized multi-omics molecular signatures to unravel the etiology of statin-induced T2D. Through systematic screening of 102 gut microbial features, 40 blood metabolites, and 131 circulating proteins in East Asians and Europeans, we identified a set of blood metabolites and proteins potentially influenced by genetically proxied statin use. Notably, Mendelian randomization analyses provided evidence that elevated circulating levels of gastric inhibitory polypeptide (GIP) were associated with an increased risk of T2D. This association between genetically proxied statin use and GIP was consistently observed across East Asian and European populations, highlighting the pivotal role of GIP in modulating the risks of statin-induced T2D. Furthermore, this study established an extensive atlas of multi-omics molecular signatures associated with statin-induced T2D, offering valuable insights for prioritizing intervention targets.

## Graphical abstract

**Figure:**
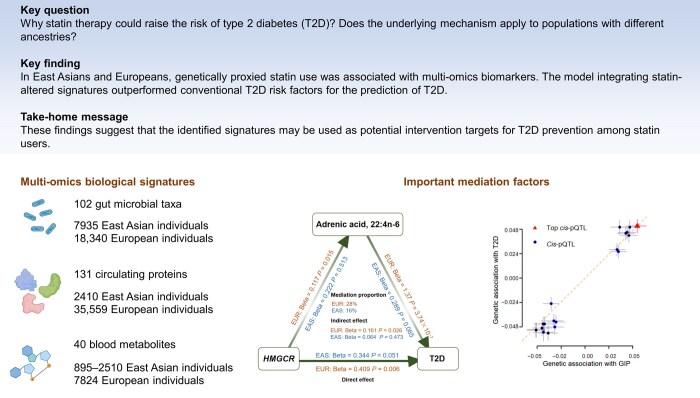

## Introduction

Statin therapy is the most potent treatment for lowering low-density lipoprotein cholesterol (LDL-C) and is widely used for primary prevention of cardiovascular diseases [1,2]. However, concerns persist about its side effects that increase the risk of type 2 diabetes (T2D). Large-scale population-based observational studies have unveiled an association between statin therapy and dose-dependent reductions in insulin sensitivity and secretion [3], accompanied by high blood glucose levels [4]. A Mendelian randomization (MR) study also supports a positive association between statin use and risk of T2D [5], which has been further confirmed in a meta-analysis of randomized controlled trials (RCTs) [6]. Despite these findings, the underlying mechanisms remain poorly understood.

Statins may have diverse influences on the biological system, as reflected by the changes in different omics signatures. For example, statins could alter the gut microbiome composition, fecal bile acids, and blood metabolites in animal models [7,8]. A small-population-based observational study suggests that statin therapy is associated with the modification of the gut microbiome composition [9]. Statin therapy is also linked to changes in blood metabolites, such as lower levels of docosahexaenoic acid [10]. To date, studies regarding the molecular signatures of statin use are limited to individual omics in a single observational cohort with small sample sizes, underscoring the need for a holistic blueprint of multi-omics constellations.

Integration of human genetic data with multi-omics signatures enables a comprehensive investigation into the effects of genetically predicted drug targets on biological systems and disease pathways [11,12]. These studies mimic the desired drug response using genetic instruments within or near the genes encoding the drug target proteins [13]. The genetic variants in the hydroxymethyl glutaryl coenzyme A reductase (*HMGCR*) gene, which modulate a rate-limiting step in the biosynthesis of cholesterol, have been utilized as genetic instruments to mimic the effects of HMGCR inhibitors (statins) on complex diseases [5,11,14].

**Figure 1:**
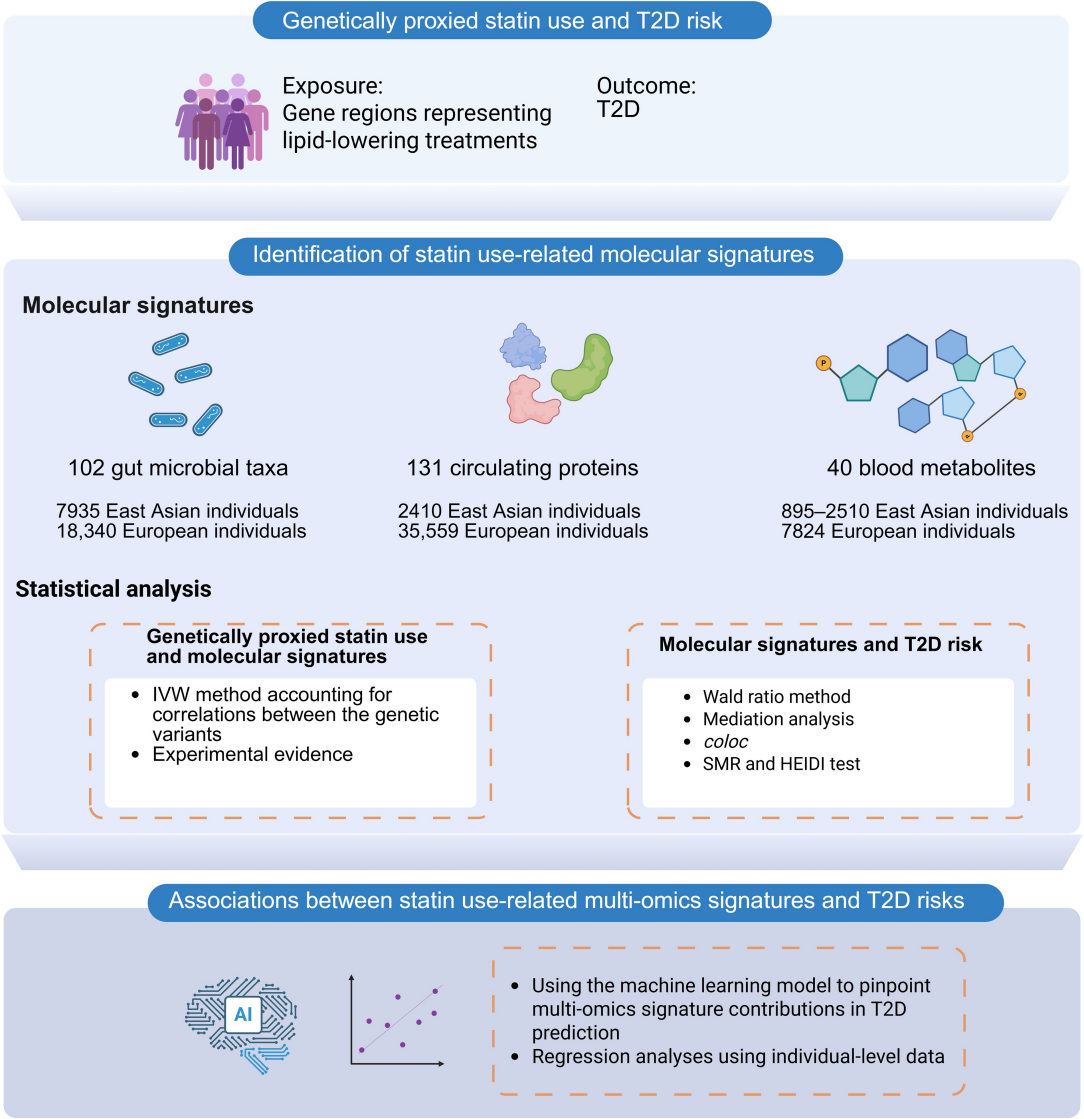
Study design for investigating cross-ethnic multi-omics signatures of statin therapy The genetic variant–LDL associations were drawn from the GLGC. The microbiome GWAS summary statistics from meta-analyses on Chinese populations and the MicroGen consortium were used to determine statin–microbiota associations. GWAS summary-level data for blood metabolites were obtained from the GNHS, TwinsUK, and KORA datasets. The genetic variant–protein associations were acquired from the GNHS, the Icelandic Cancer Project, and deCODE genetics. In the subsequent analyses, the associations between genetically proxied statin use and protein targets in the Icelandic Cancer Project and deCODE genetics were examined. Based on the individual-level GNHS data, a machine learning model was developed to prioritize targets based on feature importance in the prediction model. LDL, low-density lipoprotein; T2D, type 2 diabetes; GWAS, genome-wide association study; GLGC, Global Lipids Genetics Consortium; GNHS, Guangzhou Nutrition and Health Study; IVW, inverse-variance weighted; SMR, summary-data-based Mendelian randomization; HEIDI, heterogeneity in dependent instruments.

Here, we combined multi-omics features, including 102 gut microbial taxa, 40 blood metabolites, and 131 blood proteins, collected from East Asians (*n* = 895 to 7935) and Europeans (*n* = 7824 to 35,559), to identify shared and generalizable etiology of statin-induced (proxied by *HMGCR* variants) T2D across these two ethnic populations (**Figure 1**). Moreover, in a deeply phenotyped human cohort study, we used the machine learning model to validate that combining statin-altered molecular signatures outperformed traditional T2D risk factors for T2D prediction, providing potential targets for the prevention of statin-induced T2D.

## Results

### Genetically proxied statin use is positively associated with T2D risk

**Figure 2:**
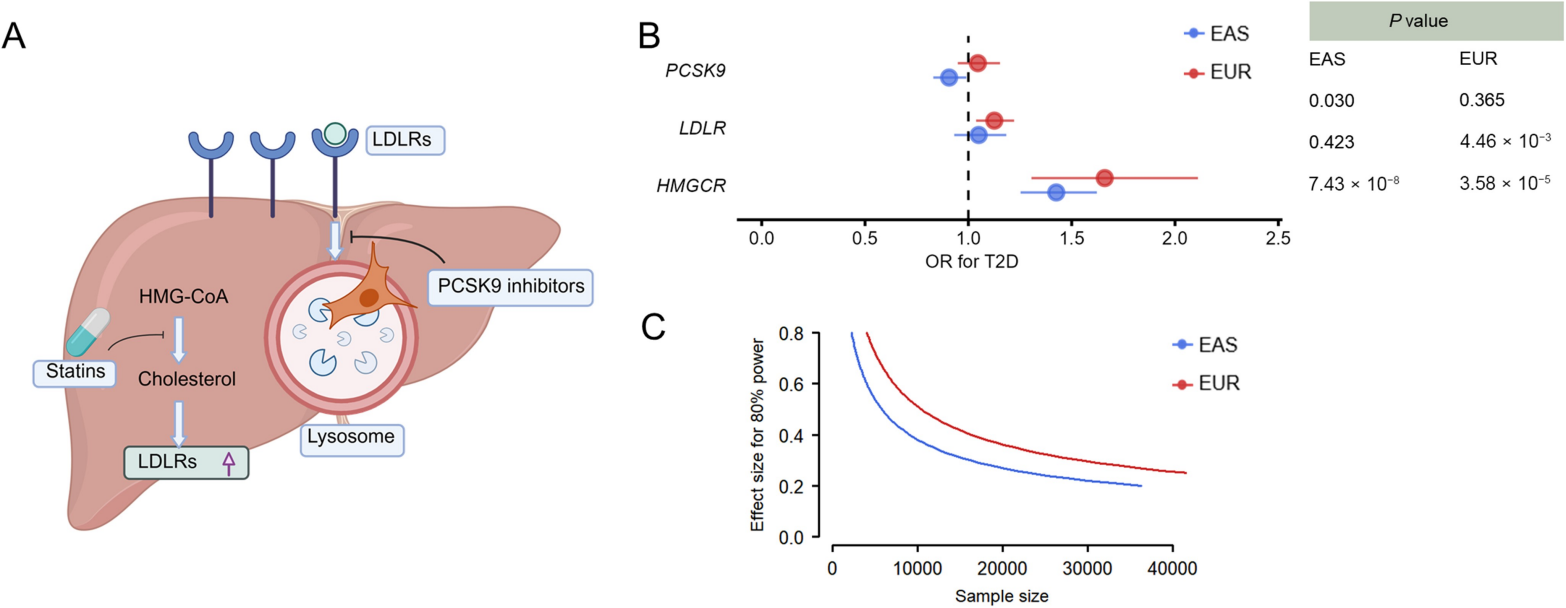
Association of lipid-lowering treatment targets with T2D and the power for MR analyses **A**. Pharmacology of statins and PCSK9 inhibitors. Statins inhibit HMGCR to prevent cholesterol production in the liver, while PCSK9 inhibitors prevent LDLRs from being degraded by PCSK9-mediated processes. **B**. Associations of LDL-related genes with T2D. The LDL-related genes, including *HMGCR*, *LDLR*, and *PCSK9*, in two populations were analyzed. **C**. Power calculations for MR analyses of the *HMGCR* gene. The plot shows the detectable estimates at 80% power. MR, Mendelian randomization; EUR, European; EAS, East Asian; HMG-CoA, hydroxymethyl glutaryl coenzyme A; HMGCR, hydroxymethyl glutaryl coenzyme A reductase; PCSK9, proprotein convertase subtilisin/kexin type 9; LDLR, low-density lipoprotein receptor; OR, odds ratio.

Previous studies have consistently affirmed a positive correlation between statin use and the risk of T2D in European populations [5,6]. The proprotein convertase subtilisin/kexin type 9 (PCSK9) inhibitors are another class of widely used lipid-lowering agents. Therapies targeting PCSK9, block the interaction between PCSK9 and low-density lipoprotein receptor (LDLR), thereby attenuating LDLR degradation. We further compared the associations of PCSK9 inhibitors and statin therapy with T2D across populations with diverse ancestries employing genetic instruments [15–17]. We performed clumping based on genome-wide association study (GWAS) summary statistics of LDL-C, using a threshold of *P* < 1 × 10^−8^ and linkage disequilibrium (LD) < 0.3 for loci within ±1000 kb of the transcription start sites of the *HMGCR*, *LDLR*, and *PCSK9* genes. The inverse-variance weighted (IVW) method, accounting for SNP correlation, was used to investigate the effect of genetically predicted lipid-lowering treatments. The effect size estimates for lipid-related genetic variants were scaled to a one standard deviation (SD) decrease in LDL-C. Our findings confirmed that statin use, represented by *HMGCR* variants, exhibited a positive association with T2D risk in both East Asian and European ethnic populations. The odds ratio (OR) was 1.43 [95% confidence interval (CI) = 1.25–1.62] for East Asians and 1.66 (95% CI = 1.31–2.11) for Europeans (**Figure 2**; Table S3).

Additionally, we extended our investigation to two other drug targets, LDLR and PCSK9, to compare the potential impacts of alternative lipid-lowering treatments on T2D. Genetic variants within the genes encoding these lipid-lowering drug targets served as instrumental variables. Results for *PCSK9* revealed inconsistency between the two populations (OR = 0.91, 95% CI = 0.83–0.99 for East Asians; OR = 1.05, 95% CI = 0.95–1.15 for Europeans). Regarding *LDLR*, the effect estimates exhibited the same direction across both populations (OR = 1.05, 95% CI = 0.93–1.18 for East Asians; OR = 1.13, 95% CI = 1.04–1.22 for Europeans), although they were notably weaker than those observed for *HMGCR*.

The consistent effect of genetically proxied statin use on increased T2D risk observed in both populations raised our interest in the shared underlying mechanisms. We systematically screened multiple microbial taxa, metabolites, and proteins that overlap between the two populations. Concurrently, the MR approach was employed to mitigate biases arising from confounding factors and bolster causal inference, mimicking an RCT.

### Gut microbial signatures of statin therapy across East Asian and European populations

**Figure 3:**
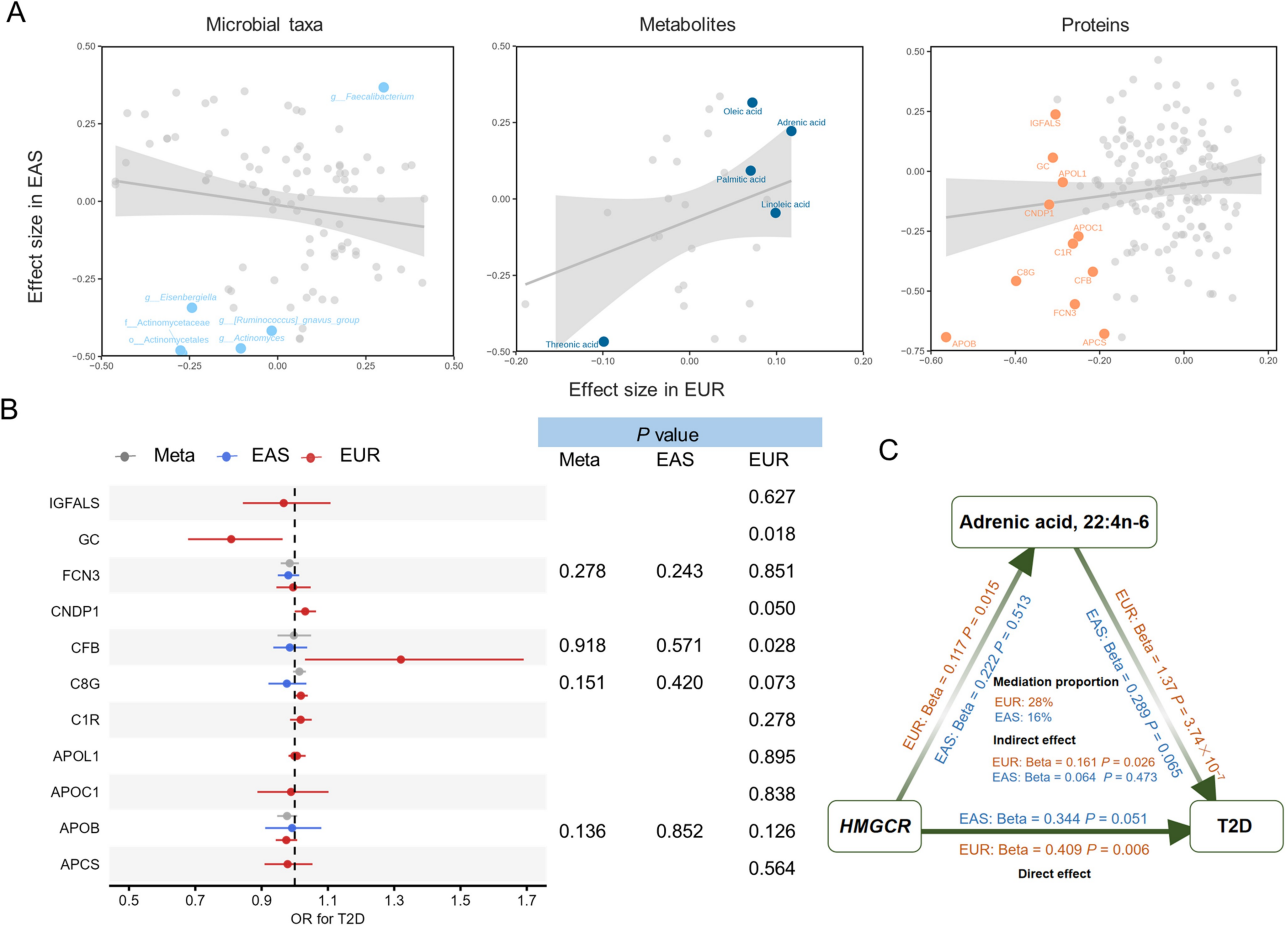
Association of genetically proxied statin use with gut microbiota, blood metabolites, and proteins **A**. Potential causal effects of genetically proxied statin use on gut microbial taxa, blood metabolites, and proteins. Results from EAS and EUR populations are shown, with a nominal significance highlighted in the fixed-effect meta-analysis. **B**. Univariable MR analyses for proteins with available genetic instruments in the corresponding populations. **C**. Further multivariable MR analysis of adrenic acid.

We examined the associations between the *HMGCR* gene region and the gut microbiome among 7935 Chinese and 18,340 Europeans [18] by analyzing 102 overlapping taxa across the two ethnic datasets (6 phyla, 9 classes, 11 orders, 22 families, 54 genera) from 16S rRNA sequencing data (Figure 1). After multiple testing correction based on microbial taxonomic levels, we did not observe significant results [false discovery rate (FDR) < 0.05]. Genetically proxied statin use showed a modest negative correlation with gut microbiota in both cohorts (Pearson correlation = −0.11). Notably, genetically proxied statin use was positively associated with the relative abundance of the genus *Faecalibacterium* in both populations (Beta = 0.36, 95% CI = 0.03–0.71 for East Asians; Beta = 0.30, 95% CI = 0.002–0.60 for Europeans). Meta-analysis suggested that genetically proxied statin use was negatively associated with the order Actinomycetales, the family Actinomycetaceae, and the genera *Actinomyces*,* Ruminococcus gnavus group*,* Eisenbergiella*, and *Erysipelatoclostridium* at a nominally significant level (*P* < 0.05) (**Figure 3**A; Table S4). The association with the order Actinomycetales remained significant after FDR correction (FDR < 0.05).

### Genetically proxied statin use has a profound impact on fatty acid metabolism

We subsequently sought to identify blood metabolites that statin therapy may influence among up to 2510 Chinese and 7824 Europeans [19]. We included 40 blood metabolites in the analysis, featuring 10 bile-acid-related metabolites and 10 long-chain fatty acids. Genetically proxied statin use positively correlated with blood metabolites in both populations (Pearson correlation = 0.27), while no significant results were observed after multiple testing correction (FDR < 0.05). At nominal significance (*P* < 0.05), the MR results showed that genetically proxied statin use was positively associated with adrenic acid (22:4n-6), oleic acid (18:1n-9), linoleic acid (18:2n-6), and palmitic acid in both populations (Figure 3A; Table S5), whereas it was negatively associated with creatine, glycocholic acid, and threonic acid across the two ethnic groups.

We further found that, in the univariable MR analysis, adrenic acid was positively associated with higher T2D risk across ethnic populations (OR = 1.29, 95% CI = 1.07–1.55 for East Asians; OR = 2.28, 95% CI = 1.51–3.44 for Europeans; meta-analysis: OR = 1.42, 95% CI = 1.19–1.68) (Table S6). The multivariable MR analysis adjusting for the genetic variants in the *HMGCR* gene region showed a concordant result with the univariable MR (Figure 3C). Mediation analysis revealed that adrenic acid was a putative mediator through which statin therapy increased T2D risk (see Materials and methods), and the proportion of mediation effect was 16% in East Asians and 28% in Europeans (Figure 3C).

### Blood proteomic signatures underlie the positive link between statin use and T2D risk

To explore proteins modulated by statin use, blood proteomics GWAS summary statistics from our public dataset of Chinese participants (*n* = 2410) [20] and published summary statistics of Europeans (*n* = 35,559) were utilized, examining 131 overlapping proteins. The results revealed a negative correlation between genetically proxied statin use and apolipoprotein B (APOB) levels, aligning with statin in lowering LDL-C (Figure 3A; Table S7). Meta-analysis of East Asian and European data showed negative associations between genetically proxied statin use and several proteins involved in the complement system, such as complement component C8 gamma chain (C8G), serum amyloid P-component (APCS), complement factor B (CFB), and complement C1r subcomponent (C1R), which are important components in the complement activation, with C8G remained significant after multiple testing correction (FDR = 0.038). MR analysis in the European population showed that genetically proxied statin use was negatively associated with vitamin D-binding protein (VDBP/GC) levels (Beta = −0.31, 95%CI = −0.56 – −0.07). GC was involved in the transport and storage of vitamin D, and it was negatively associated with T2D risk in subsequent univariable MR analysis (OR = 0.81, 95% CI = 0.68–0.96) (Figure 3B; Table S6).

**Figure 4:**
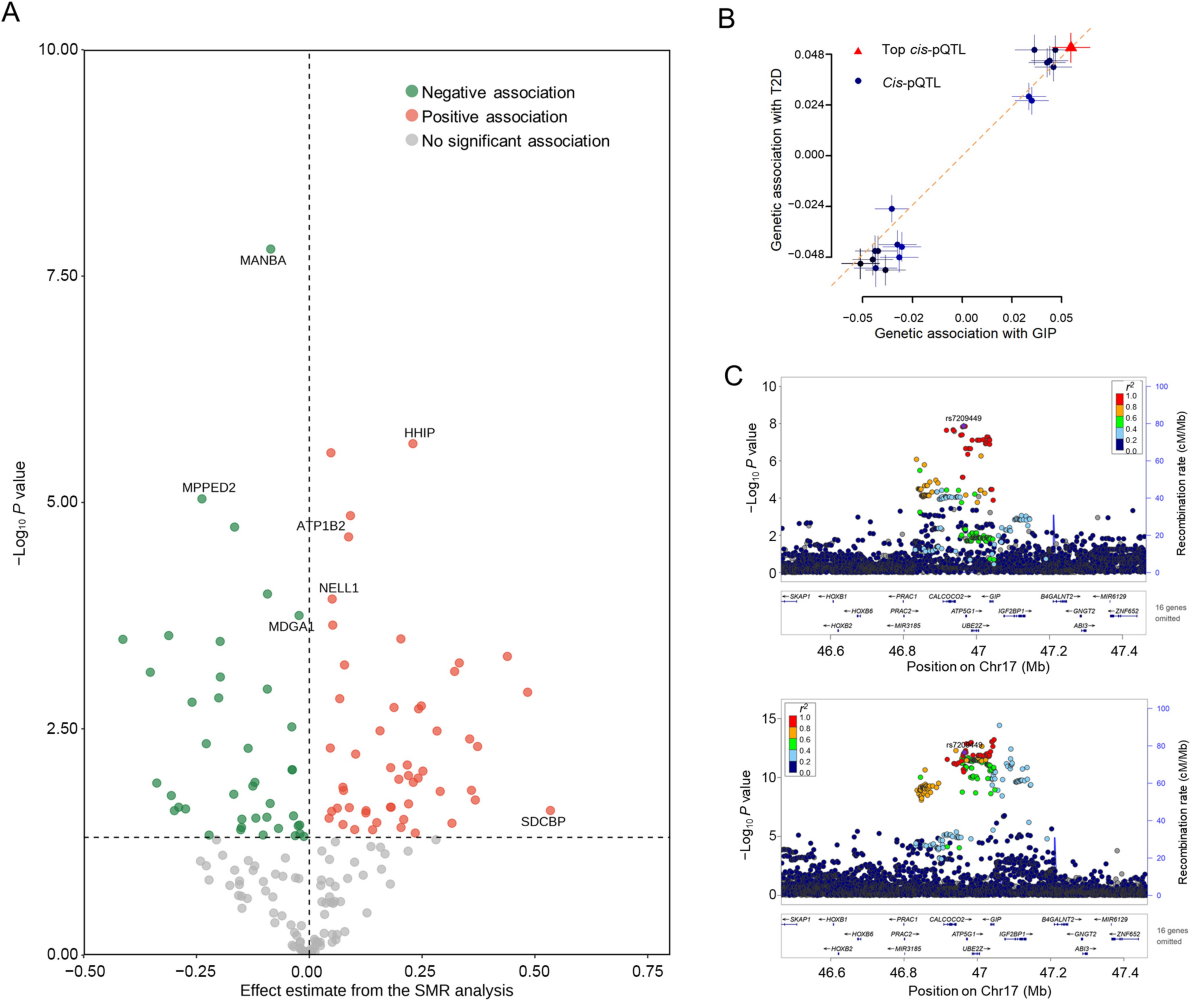
Link between genetically proxied statin use and proteomic pattern prioritizes potential therapeutic targets **A**. Proteins nominally associated with statins. Red dots denote proteins positively associated with T2D risk; green dots signify proteins negatively associated with T2D risk. Proteins that passed the HEIDI test and received support from the *coloc* method were highlighted. **B**. Associations of GIP with T2D. **C**. Colocalization analysis of GIP with T2D. The upper panel shows the GWAS results for GIP protein abundance within the GIP gene region on Chr17, while the lower panel displays the T2D GWAS results. The posterior probability of a shared causal variant for GIP and T2D (PP_4_) is 0.74, suggesting that GIP may mediate statin-induced T2D. Chr, chromosome; GIP, gastric inhibitory polypeptide; pQTL, protein quantitative trait locus.

To comprehensively understand statin-induced T2D, we assessed 4752 protein targets using the GWAS summary statistics from individuals of European ancestry. Among these, 151 proteins exhibited nominal associations with T2D risk (*P*-_IVW_ < 0.05 or *P*-_SMR_ < 0.05; SMR, summary-data-based Mendelian randomization) (**Figure 4**A; Table S8). However, the majority of proteins were regulated by a single locus, potentially resulting in a pleiotropic effect. To address this concern, we applied colocalization analysis to distinguish causality from pleiotropy [21,22]. The analysis included SNPs located within ±1000 kb of each protein-coding gene’s transcription start site. Among the 151 proteins, 12 showed associations with T2D, supported by evidence from colocalization analysis [posterior probability for H4 (PP_4_) > 0.7]. To assess whether the two traits share a common genetic signal, we performed heterogeneity in dependent instruments (HEIDI) tests [21]. The null hypothesis of the HEIDI test posits that a single causal variant underlies both the protein expression levels and the clinical trait. Eight out of the twelve proteins successfully passed the test (HEIDI *P* > 0.05). Notably, gastric inhibitory polypeptide (GIP) emerged as a protein with converging evidence from both MR analyses and two colocalization methods (Table S9).

Genetic evidence from colocalization analyses suggested GIP as a putative risk factor for T2D (OR = 2.75, SMR-multi *P* = 7.5 × 10^−6^, HEIDI *P* = 0.31, PP_4_ = 0.74) (Figure 4B and C), aligning with previous observational studies [23]. Furthermore, we sought to validate whether the associations between genetically proxied statin use and GIP levels were consistent across different populations, and confirmed positive associations using GWAS summary data of East Asians (*n* = 262) from the UK Biobank study (Beta = 1.69, 95% CI = −0.27–3.66, *P* = 0.09). Given the limited sample size, we further validated these findings within *in vitro* experiments. Leveraging published data from the Gene Expression Omnibus database (GEO: GSE8686) [24], we observed that atorvastatin treatment in human microvascular endothelial cells induced a twofold increase in GIP expression compared to untreated cells (two-sided *P* = 0.01), suggesting that statin treatment enhances GIP expression levels. The addition of mevalonate reversed the atorvastatin-induced change (atorvastatin + mevalonate-treated *vs*. vehicle-treated, *P* = 0.59), consistent with statin effects mediated via the inhibition of the mevalonate pathway [24,25]. In summary, both genetic evidence across diverse ancestries and *in vitro* experiments strongly suggest that statin use may enhance GIP levels. Our analyses, in conjunction with previous studies, affirm that circulating GIP abundance is a putative risk factor in T2D progression.

### Reverse MR analysis reveals the effect of T2D on molecular signatures

To explore how disease progression may influence molecular phenotypes and lead to reverse causal effects, we investigated the potential causal effects of genetically determined T2D risk on molecular signatures by reverse MR analysis. The genetic instruments (LD < 0.05, *P* < 5 × 10^−8^) were selected from T2D GWAS studies, including the DIAMANTE meta-analysis (East Asians: 74,124 cases and 824,006 controls) and the AGEN-T2D study (Europeans: 77,418 cases and 356,122 controls) [16,17]. Molecular signature data were derived from the same datasets used in the statin-related analyses. We applied the generalized summary-data-based Mendelian randomization (GSMR) method and the HEIDI test to assess associations [26]. In East Asian and European populations, we identified 1 and 13 gut microbial features potentially affected by genetically determined T2D risk (*P* < 0.05) (Table S10), respectively; none of these features remained significant after FDR correction on microbial taxonomic levels. For metabolites, one in East Asian and seven in European populations were potentially influenced by genetically determined T2D risk (*P* < 0.05), among which six associations in Europeans remained significant after FDR correction. None of these six metabolites was associated with genetically proxied statin use in the MR analyses (*P* > 0.05) (Table S10). Moreover, genetically determined T2D risk was associated with 4 and 97 plasma proteins in East Asian and European populations, respectively. Among these, 88 associations in Europeans passed the FDR correction. Of the 88 proteins, 5 (APOL1, APOB, CNDP1, FCN3, and IGFALS) were also associated with genetically proxied statin use (*P* < 0.05) (Table S10).

### Synergetic statin-induced multi-omics signatures enhance T2D prediction

**Figure 5:**
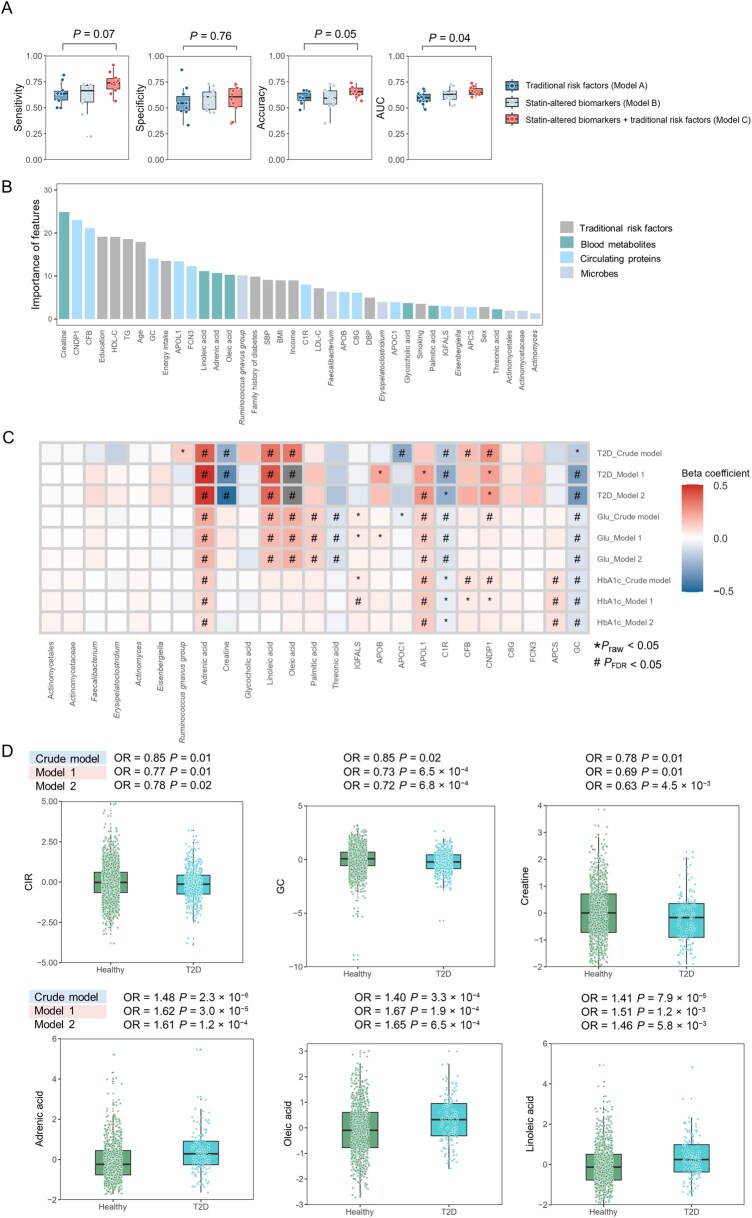
Prediction models integrating identified omics features and their contribution to T2D prediction **A**. Performance of prediction models. The paired *t*-test provides statistical significance between the traditional risk factor model (Model A) and the model incorporating statin-altered multi-omics features (Model C). **B**. Contribution of multi-omics and traditional risk factors to the support vector machine model. **C**. Heatmap showing associations between statin-induced multi-omics signatures (microbes, blood metabolites, and circulating proteins) and T2D or glycemic traits in GNHS. Effect sizes are represented as the beta coefficient increase per SD or log-odds unit. **D**. Differences in the levels of identified proteins and metabolites between T2D patients and healthy participants from GNHS. Crude model includes age, sex, and BMI; model 1 adds alcohol drinking status, smoking, energy intake, SBP, DBP, family history of diabetes, education levels, income levels, physical activity, and use of medication for T2D or dyslipidemia; model 2 uses model 1 with the exclusion of participants who took medication for T2D or dyslipidemia. AUC, area under the curve; TG, triglycerides; HDL-C, high-density lipoprotein cholesterol; LDL-C, low-density lipoprotein cholesterol; BMI, body mass index; SBP, systolic blood pressure; DBP, diastolic blood pressure; Glu, fasting glucose; HbA1c, hemoglobin A1c; SD, standard deviation; FDR, false discovery rate.

To demonstrate the clinical relevance of the identified multi-omics signatures and prioritize their importance in predicting T2D, we employed a machine learning model (support vector machine), leveraging its robustness to overfitting and kernel-based capacity to model nonlinear relationships and complex correlations in high-dimensional data. Our analyses were conducted in the Guangzhou Nutrition and Health Study (GNHS), a community-based deep phenotyping cohort study in Guangzhou, China (Table S11). We included 130 T2D cases and 130 controls randomly selected from 719 controls. The model was trained using statin-altered features evaluated in the GNHS study (*P*-_Meta_ < 0.05), including 7 microbial taxa, 7 metabolites, and 11 proteins, as well as traditional T2D risk factors including age, sex, body mass index (BMI), smoking status, LDL-C, high-density lipoprotein cholesterol (HDL-C), triglycerides (TG), systolic blood pressure (SBP), diastolic blood pressure (DBP), energy intake, family history of diabetes, education, and income. Compared with the model that only included traditional risk factors, the integration of statin-altered multi-omics features substantially improved prediction performance (**Figure 5**A). The result of the ten-fold cross-validation analysis showed higher accuracy of the integration model (mean value: 0.65 *vs*. 0.59) (Table S12) and higher area under the curve (AUC; mean value: 0.66 *vs*. 0.60).

To further demonstrate the associations between statin-induced signatures and T2D or glycemic traits, we performed a regression analysis based on cross-sectional data from the GNHS cohort. Compared with traditional risk factors in predicting T2D (Figure 5B), several identified features, such as creatine, adrenic acid (22:4n-6), and GC, were significantly associated with T2D risk (*P* values ranged from 2.3 × 10^−6^ to 0.02) (Figure 5C and D; Table S13), demonstrating a higher ranking. These findings may help interpret the statin-induced higher risk of T2D.

## Discussion

In this study, omics-wide MR analyses on 273 signatures across East Asian and European populations revealed that the *HMGCR* gene region was associated with multiple molecular biomarkers that may play critical roles in linking statin use and higher risk of T2D. The protein-wide analysis in Europeans additionally identified GIP as a potential contributor to statin-induced higher risk of T2D. To our knowledge, this is the first systematic investigation of the genetically predicted effects of statin use on multi-omics signatures and the potential biological basis of statin-induced T2D.

Statin-related proteins have been identified through several epidemiological studies [27,28]. The sample size (*n* = 135 to 225) and the number of proteins (*n* = 92 to 350) in these prior studies, however, were limited. Our study substantially expanded the number of proteins and the sample size, identifying C1R, GC, GIP, and HEXB as candidates for preventing statin-induced T2D. C1R modulates the activation of the C1 complex of the classical complement pathway [29], suggesting that the immune system and inflammation may play a role in the development of statin-induced T2D. GC is mostly localized in the liver and alpha cells, and loss of GC leads to smaller, more hyperplastic alpha cells that produce less glucagon in response to low glucose levels [30]. GIP is a hormone secreted by enteroendocrine cells in the gastrointestinal tract [31,32]. Disruption of GIP may lead to dysregulation of the enteroinsular axis, further resulting in insulin resistance and obesity. Higher levels of GIP have been observed in diabetic populations [23], and GIP levels have also been positively associated with all-cause mortality [33]. Our *in vitro* experimental results suggest that the increased effect of statin therapy on GIP may be mediated by the mevalonate pathway, with cholesterol as one downstream product. We hypothesize that reducing cholesterol or other metabolites of the mevalonate pathway may decrease dipeptidyl-peptidase-4 (DPP-4) activity. DDP-4 is a widely expressed enzyme involved in GIP degradation; therefore, the reduction in DPP-4 activity could subsequently lead to an elevation in GIP levels. This hypothesis is partially supported by the observed negative correlation between GIP and LDL-C in a population study [34]. GIP also participates in the gut-bone axis and inhibits bone resorption [32,35], aligning with the protective effect of low-dose statin treatment on bone health [36]. Statin-induced changes in GIP levels may play a significant role in human health. Collectively, these results offer potential repurposing prospects for preventing T2D among patients with statin therapy.

We identified polyunsaturated fatty acids (PUFAs) as candidate mediators for statin-induced T2D. Our data showed that statin use might increase levels of adrenic acid (22:4n-6) and linoleic acid (18:2n-6) in the two ethnic populations. Consistent with our findings, previous studies have reported that patients taking statins exhibit lower plasma levels of n-3 fatty acids or a reduced n-3 to n-6 PUFA ratio [37,38]. The possible mechanism may be that *HMGCR* could regulate key regulatory enzymes involved in the endogenous synthesis of PUFAs, including fatty acid desaturases and fatty acid elongases encoded by *ELOVL* genes [39]. We also found that genetically proxied statin use showed putative positive relationships with blood palmitic acid and oleic acid, but putative negative associations with taurodeoxycholic acid, threonic acid, creatine, and glycocholic acid. Palmitic acid and creatine have been linked to T2D or glycemic traits in previous human studies [40,41], supporting our findings. The negative association between threonic acid and glycemic traits was also found in a rodent model [42]. As threonic acid is a breakdown product of ascorbic acid, diminished threonic acid levels embody compromised ascorbic acid metabolism [43], which is suggestive of oxidative stress that plays an important role in the development of diabetes and diabetic complications [44]. Although the exact mechanism linking statins, fatty acid metabolism, and T2D remains unclear, these results highlight potential new metabolic pathways to interpret the statin–T2D association.

To address the potential reverse causation driven by disease progression, we investigated the effect of genetically determined T2D risk on molecular signatures. In the analysis of microbial taxa, metabolites, and plasma proteins, we identified five plasma proteins in the European population that were influenced by both genetically proxied statin use and genetically determined T2D risk (*P* < 0.05). Notably, the directions of effect for genetically determined T2D risk and these molecular features were opposite to those observed for genetically proxied statin use. These findings do not support the hypothesis that associations between genetically proxied statin use and these molecular traits are confounded by T2D progression.

In this study, we identified a wide range of multi-omics features associated with genetically proxied statin use. These signatures may serve as putative intervention targets to prevent T2D among statin users. Our machine learning results emphasize the importance of these biomarkers in predicting T2D, outperforming traditional risk factors. These findings, based on multi-omics data from East Asians and Europeans, reveal a potentially shared etiology for the statin–T2D association across ethnic populations. Given the high global prevalence of hyperlipidemia and the cost-effectiveness of this class of lipid-lowering medications, even modest reductions in T2D risk by intervening on these biomarkers could help alleviate the T2D pandemic.

There are several limitations to the present study. First, we use *HMGCR* variants to mimic the effect of statins, which may not be a perfect proxy, although this has been widely used previously [5,11,14]. Second, we did a cross-ethnic comparison for the effect of genetically proxied statin use on multi-omics signatures, while these signatures are not fully comparable between datasets from the two ethnic groups, with only a small proportion of overlapping biomarkers, which may bias results toward overlapping data. Third, due to the limited number of cases, we used a cross-validation strategy to assess the prediction performance of the multi-omics biomarkers within the GNHS cohort, limiting the clinical relevance of the model to predict T2D risk. External datasets with similar data structures are needed to establish generalizability. Therefore, the generalizability of our prediction model remains to be tested in future studies.

## Conclusion

In summary, the cross-ethnic evidence suggests that statin therapy affects molecular signatures, which may potentially mediate the positive association between statin use and T2D risk. These multi-omics signatures represent potential key intervention targets to prevent statin-induced T2D in future clinical settings.

## Materials and methods

### Study design and populations

We conducted a two-sample MR analysis to investigate multi-omics signatures of statin therapy. The genetic variant–LDL associations were drawn from the Global Lipids Genetics Consortium (GLGC) [15]. We analyzed 373 overlapping features (102 microbial features, 40 blood metabolites, and 131 circulating proteins) available and shared across the two cohorts of East Asian and European populations. The outcome datasets for MR analyses conducted in Europeans were derived from the following studies: the MiBioGen consortium for gut microbiome (*n* = 18,340) [18], the TwinsUK and KORA datasets for serum metabolites (*n* = 7824) [19], the Icelandic Cancer Project and deCODE genetics for plasma proteomics (*n* = 35,559) [45], and the DIAMANTE meta-analysis (74,124 cases and 824,006 controls) for T2D [16]. For East Asians, the summary statistics of serum proteomics were derived from our published GWAS data [46]. Our in-house cohort, GNHS, provided GWAS summary statistics data for other multi-omics features (*n* = 895 for serum metabolites, *n* = 2510 for erythrocyte membrane fatty acids, and *n* = 7935 for gut microbiota), while the AGEN-T2D study offered publicly available GWAS summary statistics data on T2D (77,418 cases and 356,122 controls) [17].

Furthermore, based on the individual-level data collected in our GNHS study, we developed a machine learning model to prioritize multi-omics targets according to the feature importance in the prediction model.

### Multi-omics measurement in the GNHS

The individual participant data were based on the GNHS, involving middle-aged and elderly participants living in Guangzhou, China [47]. Collection of biological samples and questionnaires from each participant was completed upon their entry into this study (2008–2013) and during follow-up visits (every three years after their recruitment). The total energy intake was estimated using data from the food frequency questionnaire (FFQ) based on habitual dietary intake over the past 12 months [48] and calculated using the Chinese Food Consumption Table (2002 edition) [49]. For analysis, energy intake was categorized into quartiles. All participants were required to fast overnight before providing their whole blood samples. Multi-omics measurements (metabolome, proteome, genome-wide genotyping, and gut microbiome) are described in File S1.

### MR analysis

We first assessed the genetically determined associations of statin therapy and two other lipid-lowering treatments (targeting LDLR and PCSK9) with T2D risk. We applied the IVW method, accounting for LD among SNPs, to investigate the effects of genetically predicted lipid-lowering treatments on T2D risk. The genetic instruments were drawn from the GLGC [15] and clumped at *r*^2^ < 0.3. LD was estimated in 494 unrelated East Asians and 495 unrelated Europeans from the 1000 Genomes Project Phase 3 [50]. The effect size estimates for lipid-related genetic variants were scaled to a one SD decrease in LDL-C. The MR analyses were performed using the “correl” option in the MendelianRandomization package [51].

HMGCR, the target of statins, was selected for future investigations to mimic the effects of statin treatment on multi-omics signatures. As described above, we used the genetic variants in the *HMGCR* gene region to represent the genetically proxied statin therapy. The IVW approach, integrating the LD matrix when estimating the coefficients, was used to assess the effect of genetically predicted statin use on multi-omics signatures. To avoid violating the MR assumption that instrumental variables are not directly associated with outcomes, we removed the genetic instruments that were significantly associated with outcomes (*P* < 5 × 10^−8^). In addition, we used the MR–Egger regression to detect the pleiotropic effects or to assess whether the genetic instruments were related to unmeasured confounders of the exposure–outcome association. Cochran’s Q statistic was used to assess the heterogeneity among genetic variants. Further power calculations for MR analyses were conducted with previously published methods [52].

### Putative causal associations between statin (*HMGCR*)-related molecular signatures and T2D risk

For the identified statin-associated proteins, previously reported protein quantitative trait loci (pQTLs) were used as instrumental variables [20,45]. Specifically, for East Asian populations, *cis-*pQTLs were clumped at a significance threshold of 5 × 10^−8^ and *trans-*pQTLs at 1.6 × 10^−10^ (*i.e.*, 5 × 10^−8^/304 proteins). Considering the biological plausibility of *cis*-regulatory loci, we used the genome-wide significance threshold for *cis*-acting loci. For European populations, all pQTLs were clumped at a significance threshold of 1.8 × 10^−9^. For statin-related blood metabolites, adrenic acid (22:4n-6) had the genetic instruments reported by Hu et al. in East Asians [53] and Guan et al. in Europeans [54]. For statin-related gut microbes, there were no available valid instrumental variables for the downstream analysis. We used the Wald ratio method to estimate the effect of the metabolites or proteins on T2D. We also performed a multivariable analysis with the adjustment of the genetic variants in the *HMGCR* gene region. The coefficient of indirect effect was estimated with the “product of coefficients” [55], and the standard errors were derived from the formula $\sigma_{\alpha \beta }=\sqrt{\alpha^{2}\sigma_{\beta }^{2}+\beta^{2}\sigma_{\alpha }^{2}-\sigma_{\alpha }^{2}\sigma_{\beta }^{2}}$.

### Reverse MR analysis

We clumped the T2D-related genetic instruments (LD < 0.05, *P* < 5 × 10^−8^) from the following studies: the DIAMANTE meta-analysis (74,124 cases and 824,006 controls) for East Asians [16] and the AGEN-T2D study (77,418 cases and 356,122 controls) for Europeans [17]. The outcome datasets were the same as those used in the molecular trait analyses related to statin use. To improve computational efficiency and account for pleiotropic effects, we employed the GSMR method and HEIDI test for large-scale reverse MR analyses [26]. We reported results with *P* < 0.05 and highlighted associations with FDR < 0.05.

### Integration of pQTLs and T2D

We used two colocalization methods to identify putative proteins mediating the relationship between statins and T2D risk. The *coloc* method was performed to test whether the proteins and T2D shared a common genetic architecture [56]. For the colocalization analysis, we set the prior probability that a SNP is associated with protein levels of T2D risk at 1 × 10^−4^, and the prior probability that a SNP is associated with both traits at 1 × 10^−5^. Furthermore, we used the SMR and HEIDI tests to investigate the potential causal relationships [21].

### Machine learning model for T2D prediction

The analysis was based on 260 GNHS participants (130 T2D cases and 130 randomly selected controls). The model was trained with the traditional T2D risk factors, including age, sex, BMI, smoking, LDL-C, HDL-C, TG, education, SBP, DBP, total energy intake, family history of diabetes, and income, as well as statin-altered signatures measured in the GNHS, including 7 taxa, 7 metabolites, and 11 proteins. Missing values in the proteomic data were imputed using half the minimum observed value. To ensure normality, the statin-altered signature data were rank-based inverse normal transformed (RINT). The support vector machine method with ten-fold cross-validation was used to compare the multi-omics features with the traditional risk factors and capture the important features contributing to T2D risk, according to the average importance in the ten-fold cross-validation. All analyses were conducted using the R software (v4.3.3) with the *e1071* package (v1.7-14).

### Observational associations between identified molecular signatures and T2D risk

To characterize the direction of associations, based on the GNHS study (*n* = 971 to 1943), we conducted logistic regression to assess the relationships between the identified multi-omics features and T2D, and used linear regression to estimate their associations with HbA1c and fasting glucose, adjusting for potential confounding factors. We fitted three models: crude model included age, sex, and BMI; model 1 added alcohol drinking status, smoking, SBP, DBP, energy intake, family history of diabetes, education, income, physical activity, and medication for T2D or dyslipidemia; model 2 was model 1 with the exclusion of participants who took medication for T2D or dyslipidemia.

## Ethical statement

The study protocol was approved by the Ethics Committee of the School of Public Health at Sun Yat-sen University (Approval No. 2018048) and the Ethics Committee of Westlake University (Approval No. 20190114ZJS003), China. All participants provided written informed consent.

## Supplementary Material

qzaf101_Supplementary_Data

## Data Availability

A website developed for querying MR estimates of the effects of statin use on molecular phenotypes is available at https://omics.lab.westlake.edu.cn/resource/statint2d.html. The raw 16S rRNA sequencing data for each cohort have been deposited in the Genome Sequence Archive [57] at the National Genomics Data Center (NGDC), China National Center for Bioinformation (CNCB) (GSA: CRA006769), and are publicly accessible at https://ngdc.cncb.ac.cn/gsa. The raw data for serum proteomes have been uploaded to iProX (ProteomeXchange: PXD039236, PXD039231, and PXD038253), and are publicly accessible at https://www.iprox.cn. Other datasets generated and analyzed in this study are available upon reasonable request by bona fide researchers for specified scientific purposes via contacting the corresponding authors.

## CRediT author statement

**Fengzhe Xu:** Methodology, Investigation, Formal analysis, Writing – original draft. **Min Yang:** Methodology, Investigation, Formal analysis, Writing – original draft. **Wei Hu:** Data curation. **Shuai Yuan:** Writing – review & editing. **Xue Cai:** Data collection. **Wanglong Gou:** Data collection. **Zelei Miao:** Data curation. **Bang-yan Li:** Data curation. **Liang Yue:** Data collection. **Zhangzhi Xue:** Data collection. **Menglei Shuai:** Data collection. **Luqi Shen:** Writing – review & editing. **Yuanqing Fu:** Writing – review & editing. **Tiannan Guo:** Conceptualization. **Yu-ming Chen:** Conceptualization. **Ju-Sheng Zheng:** Conceptualization, Supervision, Writing – review & editing. All authors have read and approved the final manuscript.

## ORCID

0000-0002-2686-1445 (Fengzhe Xu)

0009-0000-5429-9253 (Min Yang)

0000-0001-9332-9670 (Wei Hu)

0000-0001-5055-5627 (Shuai Yuan)

0000-0002-7427-2100 (Xue Cai)

0000-0002-4256-9452 (Wanglong Gou)

0000-0001-9478-1933 (Zelei Miao)

0000-0001-7446-2505 (Bang-yan Li)

0000-0001-6478-7306 (Liang Yue)

0000-0001-7311-6221 (Zhangzhi Xue)

0000-0001-9983-1477 (Menglei Shuai)

0009-0003-4349-4063 (Luqi Shen)

0000-0002-3955-9376 (Yuanqing Fu)

0000-0003-3869-7651 (Tiannan Guo)

0000-0003-1658-5528 (Yu-ming Chen)

0000-0001-6560-4890 (Ju-Sheng Zheng)
