# Supplementary material for: Cross-ethnic Molecular Signatures Underpin the Adverse Impact of Statin Use on Type 2 Diabetes
Source: Genomics Proteomics Bioinformatics. 2025 Nov 6;23(5):qzaf101. doi: 10.1093/gpbjnl/qzaf101 (PMC12975336; doi:10.1093/gpbjnl/qzaf101)
Supplement: qzaf101_Supplementary_Data [file qzaf101_supplementary_data.zip › File S1.docx]

**File S1 Supplementary methods**

**Fecal sample collection and microbiome sequencing in the GNHS**

The collected fecal samples were stored at −80°C before being analyzed. We used primers 341F (5'-CCTACGGGNGGCWGCAG-3') and 805R (5'-GACTACHVGGGTATCTAATCC-3') for amplifying 16s rRNA gene, and sequenced the amplified 16s rRNA gene with Illumina MiSeq System (Illumina Inc., CA). The subsequent merge-pair, quality filtering and analysis on amplicon sequences were performed with Quantitative Insights into Microbial Ecology (QIIME) software (QIIME2 v2019.10) [1]. We filtered and reduced the noise from marker gene Illumina sequences and chimeric sequences (“consensus”) with DADA2 algorithm, and further screened for the rare features that were detected in only one sample. We mapped to SILVA 99% OTUs reference databases [1] with a trained Naive Bayes classifier.

**Measurement of serum metabolites in the GNHS**

We performed targeted metabolomics measurements of these samples with a triple quadrupole system (ACQUITY UPLC-Xevo TQ-S, Waters Corp.). To extract serum metabolites, we vortexed the serum samples in ice-cold methanol with internal standards and derived collected the supernatant. We then diluted the sample with 50% ice-cold methanol and centrifuged the diluted sample at 4000g before extracting the supernatant containing internal standards. The extracted internal standard-containing supernatant was sealed until ready for LC-MS/MS detection. The setup of the instrument was as follows: C18 analytical column (2.1*100 mm,1.7 μM) was used for separation, and the column temperature was set to 40°C. We used water with 0.1% formic acid as mobile phases A, and a mixture of acetonitrile and IPA at a ratio of acetonitrile: IPA = 90:10. The detection and quantification of metabolites were completed at Metabo-Profile Corp. (Shanghai, China).

In addition to the above blood metabolites, we also measured blood erythrocyte fatty acid composition by gas chromatography (7890 GC, DB-23 capillary column 60m×0.25mm internal diameter×0.15μm film, Agilent, California) in form of ratios (%) of total fatty acids. Fatty acids were identified and measured with commercial standards (Nu-Chek Prep, Minnesota).

**Serum proteomics profiling in the GNHS**

Peptides were purified from the serum samples as described previously [2,3]. In brief, 1 µL of serum samples were digested with 20 µl of lysis buffer prepared with 8 M urea (Sigma, #U1230) in 100 mM ammonium bicarbonate (ABB). Subsequently, the digestion products were reduced and alkylated in 10 mM tris (2-carboxyethyl) phosphine (TCEP, Sigma #T4708) and then alkylated in 40 mM iodoacetamide (IAA, Sigma, #SLCD4031). 70 µL of 100 mM ABB was added to the resulting reaction solution before digestion with trypsin at an enzyme/substrate ratio of 1:60 in 2 successive steps of 4 h and 12 h, respectively. The pH of the reaction was adjusted to 2-3 with 1% trifluoroacetic (Thermo Fisher Scientific, #T/3258/PB05) to quench the reaction. Peptides were further purified in C18 SOLAu columns (Thermo, #60209-001) and analyzed on MS. We then analyzed the peptide samples on a TripleTOF 5600 system (SCIEX, CA) in couple with Eksigent NanoLC 400 System (Eksigent, Dublin, CA) over a 20 min linear LC gradient by SWATH-MS as previously described [2,3]. After SWATH acquisition, we analyzed the resulting wiff files with DIA-NN (1.7.12) [4] based on 3474 peptide precursors from a serum spectral library and 536 unique proteins of Homo Sapiens from Swiss-Prot database [3]. To set up for the DIA-NN method, we set the peptide length range from 5 to 30, the precursor m/z range from 400 to 1200, and the fragment ion m/z range from 100 to 1500. We set the retention time extraction window by default and then m/z extraction window at 20 ppm for MS1 and 50 ppm for MS2. The FDR for protein and peptide was controlled below 1%.

**Genotyping in the GNHS**

DNA was separated from leukocytes with TIANamp® Blood DNA Kit. We measured DNA concentrations via the Qubit quantification system (Thermo Scientific, Wilmington, DE) and applied Illumina ASA-750K arrays for genotyping. SNPs with HWE P-value<0.00001 and missing call rate > 0.05 were removed. We removed individuals with a high or low ratio of heterozygous genotypes (3 standard deviations from the mean), sex mismatch, or distinct ancestries (the top two principal components 5 standard deviations from the mean). Subsequently, we conducted genetic variants mapping to the 1000 Genomes Project Phase3 v5 with SHAPEIT [5] and imputation with Minimac3 [6]. Genetic variants with RSQR > 0.3 were kept for the following GWAS analysis.

**Genome-wide association analyses in the Chinese participants**

The abundances of multi-omics features were transformed using methods consistent with the GWAS analyses in Europeans. In brief, gut microbial taxa present in more than 90% of participants were included. The abundances of microbial metabolites were natural log-transformed and standardized to one standard deviation. The protein abundance was transformed with the rank-based inverse normalization transformation. We used the mixed linear model with GCTA (version 1.93.3) to assess the linear associations between genotypes and microbiota, metabolites as well as proteins. The covariates adjusted in the model were age, sex, and the top 10 genetic principal components of ancestry for gut microbiota, the top 5 genetic principal components of ancestry for metabolites and proteins.

Our GWAS summary statistics of Chinese (representing East Asians) are based on our GWAS of these omics data from the GNHS including proteomics [7], gut microbiome [8], and serum metabolites. For the gut microbiome, we additionally included data from other four cohorts (Zhejiang Metabolic Syndrome Cohort (*n* = 1151), Tongji-Shuangliu Birth Cohort (*n* = 4455), Westlake Precision Birth Cohort (*n* = 453), and Westlake Personalized Nutrition and Health Cohort for Drug Addicts (*n* = 193); for metabolites, we additionally included erythrocyte membrane fatty acids (myristic acid (14:0), palmitic acid (16:0), palmitoleic acid (16:1n7), stearic acid (18:0), oleic acid (18:1n9), linoleic acid (18:2n6), arachidonic acid (20:4n6), and adrenic acid (22:4n6)) measured in the GNHS study [9].

Reference

[1] Bolyen E, Rideout JR, Dillon MR, Bokulich NA, Abnet CC, Al-Ghalith GA, et al*.* Reproducible, interactive, scalable and extensible microbiome data science using QIIME 2. Nat Biotechnol 2019;37:852–7.

[2] Gou W, Yue L, Tang XY, Wu YY, Cai X, Shuai M, et al*.* Circulating proteome and progression of type 2 diabetes. J Clin Endocrinol Metab 2022;107:1616–25.

[3] Zhang Y, Cai X, Ge W, Wang D, Zhu G, Qian L, et al*.* Potential use of serum proteomics for monitoring COVID-19 progression to complement RT-PCR detection. J Proteome Res 2022;21:90–100.

[4] Demichev V, Messner CB, Vernardis SI, Lilley KS, Ralser M. DIA-NN: neural networks and interference correction enable deep proteome coverage in high throughput. Nat Methods 2020;17: 41–4.

[5] Delaneau O, Howie B, Cox AJ, Zagury JF, Marchini J. Haplotype estimation using sequencing reads. Am J Hum Genet 2013;93:687–96.

[6] Das S, Forer L, Schönherr S, Sidore C, Locke AE, Kwong A, et al*.* Next-generation genotype imputation service and methods. Nat Genet 2016;48: 1284–7.

[7] Xu F, Yu EY, Cai X, Yue L, Jing LP, Liang X, et al*.* Genome-wide genotype-serum proteome mapping provides insights into the cross-ancestry differences in cardiometabolic disease susceptibility. Nat Commun 2023;14:896.

[8] Xu F, Fu Y, Sun TY, Jiang Z, Miao Z, Shuai M, et al*.* The interplay between host genetics and the gut microbiome reveals common and distinct microbiome features for complex human diseases. Microbiome 2020;8:145.

[9] Miao Z, Lin JS, Mao Y, Chen GD, Zeng FF, Dong HL, et al*.* Erythrocyte n-6 polyunsaturated fatty acids, gut microbiota, and incident type 2 diabetes: a prospective cohort study. Diabetes Care 2020;43:2435–43.
