# Supplementary material for: Cross-ethnic Molecular Signatures Underpin the Adverse Impact of Statin Use on Type 2 Diabetes
Source: Genomics Proteomics Bioinformatics. 2025 Nov 6;23(5):qzaf101. doi: 10.1093/gpbjnl/qzaf101 (PMC12975336; doi:10.1093/gpbjnl/qzaf101)
Supplement: qzaf101_Supplementary_Data [file qzaf101_supplementary_data.zip › Supplementary Material Captions.docx]

**Supplementary** **material**

**File S1 Supplementary methods**

**Table S1 Genetic variants included in MR analyses for Europeans**

**Table S2 Genetic variants included in MR analyses for East Asians**

**Table S3 Estimates for the effect of genetically proxied lipid-lowering drug targets on type 2 diabetes**

**Table S4 Estimates for the effect of genetically proxied statin use on gut microbiota**

**Table S5 Estimates for the effect of genetically proxied statin use on blood metabolites**

**Table S6 Genetically predicted effect of identified features on type 2 diabetes**

**Table S7 Estimates for the effect of genetically proxied statin use on circulating proteins**

**Table S8 Genetically predicted protein levels and risks of type 2 diabetes**

**Table S9 Effect of genetically proxied statin use on additional protein targets in Europeans**

**Table S10 Estimates for the effect of genetically determined T2D risk on molecular signatures**

**Table S11 Characteristics of Guangzhou Nutrition and Health study**

**Table S12 Performance of the SVM prediction model**

**Table S13 Associations between identified features and glycemic traits**
